# EEG-based epileptic seizure detection using binary dragonfly algorithm and deep neural network

**DOI:** 10.1038/s41598-023-44318-w

**Published:** 2023-10-18

**Authors:** G. Yogarajan, Najah Alsubaie, G. Rajasekaran, T. Revathi, Mohammed S. Alqahtani, Mohamed Abbas, Madshush M. Alshahrani, Ben Othman Soufiene

**Affiliations:** 1 Department of Information Technology, Mepco Schlenk Engineering College, Sivakasi, Tamil Nadu 626005 India; 2 Department of Computer Sciences, College of Computer and Information Sciences, Princess Nourah bint Abdulrahman University (PNU), P.O. Box 84428, 11671 Riyadh, Saudi Arabia; 3 Radiological Sciences Department, College of Applied Medical Sciences, King Khalid University, 61421 Abha, Saudi Arabia; 4 BioImaging Unit, Space Research Centre, University of Leicester, Michael Atiyah Building, Leicester, LE1 7RH UK; 5 Electrical Engineering Department, College of Engineering, King Khalid University, 61421 Abha, Saudi Arabia; 6 Department of Radiology, KMGH, Khamis Mushayt, Saudi Arabia; 7 PRINCE Laboratory Research, ISITcom, Hammam Sousse, University of Sousse, Sousse, Tunisia

**Keywords:** Diseases, Health care, Energy science and technology

## Abstract

Electroencephalogram (EEG) is one of the most common methods used for seizure detection as it records the electrical activity of the brain. Symmetry and asymmetry of EEG signals can be used as indicators of epileptic seizures. Normally, EEG signals are symmetrical in nature, with similar patterns on both sides of the brain. However, during a seizure, there may be a sudden increase in the electrical activity in one hemisphere of the brain, causing asymmetry in the EEG signal. In patients with epilepsy, interictal EEG may show asymmetric spikes or sharp waves, indicating the presence of epileptic activity. Therefore, the detection of symmetry/asymmetry in EEG signals can be used as a useful tool in the diagnosis and management of epilepsy. However, it should be noted that EEG findings should always be interpreted in conjunction with the patient's clinical history and other diagnostic tests. In this paper, we propose an EEG-based improved automatic seizure detection system using a Deep neural network (DNN) and Binary dragonfly algorithm (BDFA). The DNN model learns the characteristics of the EEG signals through nine different statistical and Hjorth parameters extracted from various levels of decomposed signals obtained by using the Stationary Wavelet Transform. Next, the extracted features were reduced using the BDFA which helps to train DNN faster and improve its performance. The results show that the extracted features help to differentiate the normal, interictal, and ictal signals effectively with 100% accuracy, sensitivity, specificity, and F1 score with a 13% selected feature subset when compared to the existing approaches.

## Introduction

Electroencephalogram (EEG) is an electrical signal produced by the human brain, which indicates the functions performed by the brain and helps to monitor the development of the brain, coma, alpha rhythm, epilepsy, Alzheimer, strokes, migraines, test drug effects, investigate mental and sleep disorders, etc. in medical healthcare. Epilepsy is one of the most common neural disorders caused due to abnormal brain activities which can be identified by a symptom called epileptic seizure which is getting more common across the world. Epilepsy detection is commonly done using continuous electroencephalography (cEEG), amplitude-integrated electroencephalography (aEEG), Magnetic resonance imaging (MRI), functional magnetic resonance imaging (fMRI), Computed tomography (CT) scan, and etc.^1^. Early detection and diagnosis of seizures using the above-mentioned techniques help to treat people with epilepsy well and that may reduce the chance of premature death. According to the World health organization (WHO), more than 50 million people across the world are affected by epilepsy currently, and also it is estimated that around 80% of the people with epilepsy live in developing countries^2^. Nevertheless, 75% of the epilepsy-affected people living in developing countries don't get treatment for epilepsy and hence experience a frequent seizure that reduces their lifetime. The analysis and diagnosis of epileptic seizures are commonly done using EEG signals by medical practitioners. The interpretation of EEG signals by expert physicians and medical practitioners requires more effort and time. An automatic seizure detection system will help the practitioners to study and analyze the EEG signal with ease, reducing their effort and time significantly. Automatic seizure detection has been done using time domain, frequency domain or time-frequency domain analysis of EEG signals in most of the conventional methods proposed so far. The seizure signal is characterized by its spikes and sharp waves (SSWs) which are unpredictable and point to abnormal neuronal activities commonly found in patients with epilepsy. Generally, SSWs are designated as interictal because they occur in between ictal (seizure) events.

**Figure 1:**
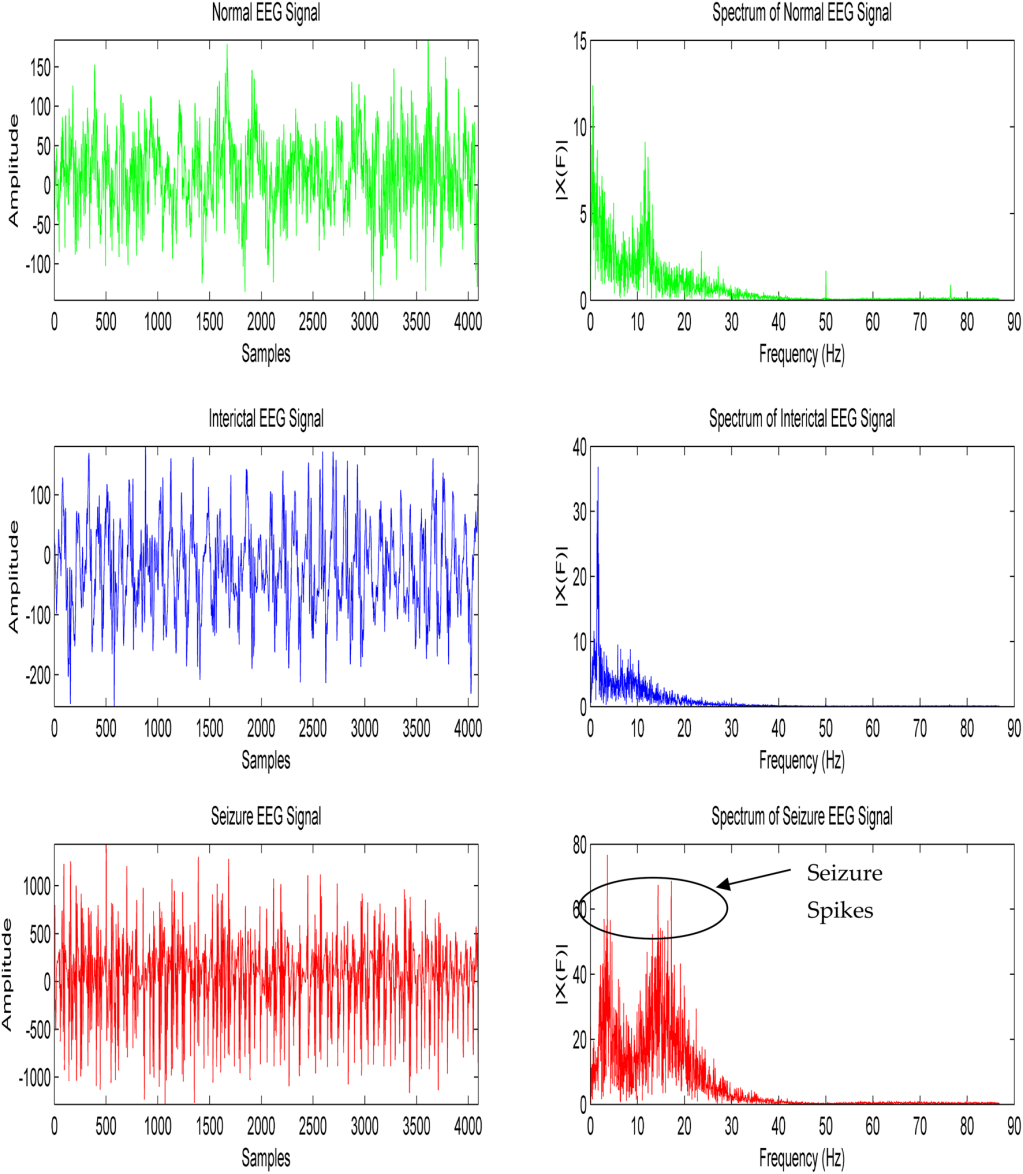
EEG Signal in Time Domain and Frequency Domain (Normal, Interictal, Seizure).

Several features that have been considered to classify these EEG signals are mobility, complexity, activity, higher order moments, probability density function parameters, and entropy, etc. The time domain and frequency domain representation of normal, interictal and epileptic seizure EEG signals has been depicted in Fig. 1. The amplitude of the EEG signals illustrated in Fig. 1 is in μVolts. Signadomainsessing techniques that explore the time domain have non-stationary EEG signals which are more popular among the seizure detection researchers’ group. The features from Wavelet transform (WT) analysis of EEG signals have shown prospective results in seizure detection. However, the performance of the wavelet-based approaches depends largely on the type of wavelet basis function and the number of levels used for EEG decomposition. The frequency domain characteristics of EEG signals (normal, interictal, seizure) that spread over different frequency bands are shown in Fig. 1. The robust selection of time domain and frequency domain features, extracted from EEG signals notably improves the efficiency of automatic epileptic seizure detection system. Hence, in this paper, we propose an efficient seizure detection system that is simple, accurate, fast, and cost-effective in nature and helps the seizure detection researchers’ community across the entire world.

The remainder of this paper is as follows: "Related work" section discusses the related works in the field of epileptic seizure detection and "Materials and methods" section describes the working of the proposed system model. "Results and discussion" section illustrates the experimental results and discussion. "Conclusion" section concludes the paper.

## Related work

EEG signals are an important source of information for the medical practitioners to analyze the activity of the brain while diagnosing patients ailing from neurological disorders. The different frequency components from EEG signals that are useful for medical analysis are as follows: Delta ($f \le 3\;{\text{Hz}}$), Theta ($3.5\;{\text{Hz}} \le f \le 7.5\;{\text{Hz}}$), Alpha ($7.5\;{\text{Hz}} < f \le 13\;{\text{Hz}}$), Beta ($13\;{\text{Hz}} < f \le 26\;{\text{Hz}}$), and Gamma ($26\;{\text{Hz}} < f \le 100\;{\text{Hz}}$). The different physical and mental activities associated with the brain have the frequency bands mentioned above. The EEG signals are time-variant and non-stationary in nature which cannot be detected easily. Time–frequency methods such as discrete wavelet transform, wavelet packet decomposition, dual-tree complex wavelet transform, empirical mode decomposition, etc. are used to extract features from it. The wavelet-based decomposition is used to detect transients, spikes, and epileptic seizures from EEG signals efficiently.

A wavelet-based method is proposed to capture the rhythmic nature of seizure discharges^3^. This method examines the fluctuation of different frequency ranges compared to the background and identifies rhythmic bursts, which are commonly found in the background, to avoid false seizure detection. This method had achieved only 87% sensitivity on average. The frequency flow dynamics have been studied using wavelets, while temporal lobe seizures generate theta waves^4^. It employs a ridge extraction algorithm to estimate the instantaneous frequency from the normalized scalogram. It was observed that, prior to seizure onset, the theta waves were built up, and the frequency increased immediately after the onset of the seizure. But, the other types of epilepsy, such as non-temporal and generalized epilepsy were not explored in the above study. A normalized wavelet-based index, named the Combined seizure index (CSI) is used for epileptic seizure detection using the scalp EEG signal^5^. A seizure alarm signal is generated with respect to the channel-based information. The advantage of this method is lateralization of the seizure focus on temporal lobe epilepsy moreover, this method is patient-specific, and it fails to consider patients with extratemporal lobe epilepsy.

A tree-based wavelet transforms and directed acyclic graph Support vector machine (SVM) have been used to extract the features from EEG signals in order to classify, whether they indicate seizure or normal^6^. It works in two stages by extracting the detailed and approximate information first, followed by a multi-label classification. The graph-based SVM, along with the extracted features, most of the discriminating features while classifying the signals. However, they have considered only accuracy as the performance metric and the false positive rate is also observed to be high. The high-frequency activity has been analyzed in the intracranial EEG of epileptic patients during seizure detection. This method employed wavelet decomposition, feature extraction, adaptive thresholding, and artifact removal with 30 hours of intracranial Scalp EEG signals^7^. The system produced reasonable results in terms of sensitivity and latency, but it fails to detect seizures with subtle or absent high frequencies. The important observation is that high frequencies in EEG signals have the potential to contribute significantly to the detection of epileptic seizures. Dual-tree complex wavelet transform (DTCWT) and Fourier features have been used for seizure detection. Fast Fourier Transform has been applied to 4th and 5th scale of DTCWT output, which is capable of 100% classification accuracy. The performance metrics considered for evaluation are accurate for CPU execution time^8^. Detailed review of applications of wavelet transform in EEG-based seizure detection and an automated computer-aided seizure detection and epilepsy diagnosis system is proposed^9^. It uses a multi-paradigm approach that integrates wavelets, non-linear dynamics, chaos theory, and neural networks. Also, it uses seven different features for the classification of EEG signals in the offline-online approach. The offline training and testing help to classify the EEG signals in offline mode.

Temporal correlation within EEG signals is exploited for better feature extraction and classification, and in turn better seizure detection^10^. Any abrupt changes in the temporal correlation within the EEG signals are effectively detected which indicates the rise of the seizure phenomenon. Two methods have been proposed to detect seizures based on temporal correlation. Intrinsic mode function (IMF) and Discrete cosine transform (DCT) have been used to extract the features followed by classification using least square SVM. This approach outperforms the state-of-the-art approaches in terms of sensitivity, specificity, and accuracy. The decisions made during the presurgical stages for pediatric patients have been made using EEG signal analysis^11^. A seizure-specific wavelet (seizlet) has been designed using four structural features which have been extracted and classified using a hybrid optimization algorithm. The seizlet kernel has been modeled to extract the seizure patterns effectively from the EEG signals than the existing methods which are observed in terms of accuracy and false positive rate. A three-band orthogonal filter bank method has been designed to detect alcoholism from the EEG signals^12^. The concepts that are involved in detecting alcoholism are duration-bandwidth product, orthogonal filter bank, log energy, and least square SVM. The features extracted using the logarithm of the energies of the wavelet sub-bands have been passed to the SVM classifier model to detect the alcoholic signals from normal Electrocardiogram (ECG) signals which assist medical practitioners during the diagnosis of alcoholic patients.

With the increase in the volume of medical records, it is not easy for medical experts to analyze the records easily and efficiently. Machine learning and deep learning algorithms help to analyze these medical data quickly and with high efficiency. Data mining concepts augmented with machine learning and deep learning help to build several types of classifiers that can classify multidimensional data with ease. A cross-correlation with an artificial reference method has been proposed to reduce the possible consequences of the random selection of signal as a reference during the classification of data. Using cross-correlation and ECG as a reference signal for the classifier improved the performance of EEG seizure detection compared to traditional classifier algorithms^13^.

A new non-parametric model based on the localization of epilepsy seizure with non-parametric tools which produces better resolution in the frequency and time domain as opposed to visual inspection of EEG rhythm^14^. Daubechies level 13 wavelet has been used to obtain the sub-bands from which the features have been extracted which improve the detection of short seizures and spikes. The evaluation metric considered in this work is sensitivity and accuracy.

Optimal Orthogonal wavelet filter banks (OWFB) have been designed to reduce the frequency spread in EEG signals during seizure detection. A Semi-definite programming (SDP) formulation has been done to design the optimal orthogonal wavelet filter^15^. The features have been obtained using the Minimally mean squared frequency localized (MMSFL) OWFB approach and tested under two different EEG datasets which detect a seizure with very high accuracy.

A multimodal seizure detection algorithm was developed considering the fast rhythmic activity and patterns captured using a graphical software tool and the quantitative information of EEG, ECG, and Electromyogram (EMG). A rule-based classifier is employed for better interpretation using which seizure detection is done in an automated manner^16^. This system involves a complex set-up that requires capturing three physiological signals namely EEG, ECG and EMG. A cross-bispectrum-based feature has been used to detect epileptic seizure activity from multi-channel intracranial EEG (iEEG) data. The cross-bispectrum features have been passed to an SVM classifier to differentiate the ictal state from the interictal state^17^. An average moving filter has been used as a post-processing method to improve the classification accuracy by smoothening which reduces the noisy behavior of the SVM output.

The characteristics of the EEG signal tend to vary with time and the state of a patient. Robust feature selection helps to reduce the volume of time used for training which makes a system powerful and works faster. The spectral content of EEG signals has been modeled as an Autoregressive (AR) model and the output of the AR model has been applied to a Multilayer Perceptron classifier to classify the seizure signals. This approach requires per-channel labeling which is difficult when the detection system is made online in real-time medical diagnosis applications^18^.

An Extreme learning machine (ELM) combined with an Optimized sample entropy (O-SampEn) algorithm to identify the seizure from EEG signals. This approach has high detection accuracy and very high computation speed, which demonstrates its huge potential for the real-time detection of epileptic seizures^19^. Teager energy cepstrum (TECEP) and pattern recognition neural networks have been used for the detection of epileptic seizure detection. The teager energy operator is characterized by time resolution that can track rapid changes in signal energy. Teager energy cepstrum involves a signal being divided into sub-bands, followed by log compression and inverse discrete cosine transform for each sub-band^20^. The features constructed using cepstrum help to discriminate the different EEG signals and provide feedback to clinical neurophysiologists. This finds its significance in applications like seizure warning/control systems and delivering abortive responses/monitoring patients using implantable therapeutic devices. A lagged-Poincare-based feature extraction scheme combined with an extreme learning machine is proposed to detect epileptic seizures. Six different metrics have been used to characterize the performance of the proposed system^21^.

A detailed study of different machine learning classifiers on the scalp EEG dataset was done. Statistical features have been extracted in the time and frequency domain and using Analysis of variance (ANOVA) most significant features have been selected^22^. The Extremely Randomized Decision Tree algorithm has been observed to produce better results in terms of accuracy, sensitivity, and specificity. A machine learning approach has been proposed based on fast and accurate detection of seizures from EEG signals. It employs discrete wavelet transform and k-nearest neighbor/deep neural network classifier for ictal detection. A prototype also has been designed using the hardware in the loop approach which helps in smart health care using the internet of medical things^23^.

A study of various machine learning classifiers and deep learning neural networks has been done on the EEG dataset for seizure forecasting. The performance of six machine learning classifiers and three deep learning networks for multi-label EEG classification has been measured in terms of precision and accuracy^24^. A machine-learning-based seizure detection system that collects EEG data from the closed loop interface implanted in the patient's brain has been proposed^25^. A set of several time domain and frequency domain features in four different categories has been extracted and applied to standard machine algorithms such as SVM, K Nearest Neighbours (KNN), and Gradient boost tree for classifying it as a seizure signal or not. A comparative study is done on the performance of traditional machine learning and deep learning algorithms in epileptic seizure detection. Karl Pearson’s coefficient of correlation is used to eliminate irrelevant attributes that contribute to the improvement in classification accuracy and speed. The ensemble and deep learning models outperform the traditional machine learning techniques in terms of accuracy. Few more modalities for automated seizure detection use various principles that includes DWT, cepstrum, machine learning, etc^26–33^.

A single-channel seizure detection system using brain-rhythmic recurrence biomarkers and an ONASNet-Based Transfer Learning have been analyzed^39^. It achieves an accuracy of 99.67% for single-channel EEGH datasets. Recurrence plots have been used as a means to capture the non-linear dynamics in the EEG signal^40,41^. Riemannian geometry has been used to transform the covariance matrices estimated from the non-invasive scalp EEG (sEEG) signals into a feature vector^42^.

Deep learning-based approaches are getting more popular in the field of medical diagnosis. Deep learning models help to predict covid-19^43^, segment cervical cytology images^44^, detect breast cancer^45^, and distance-directed target searching^46^. Though deep learning-based approaches produce interesting results and performance they need more volume of data for their learning. Handling those voluminous sensitive healthcare data whenever they are stored in the cloud creates several confidentiality and privacy issues among patients. Hence, ensuring the confidentiality of those sensitive data is also challenging nowadays^47^. Deep learning has played a significant role in forecasting new COVID-19 cases^48^ and also helped for spatiotemporal modeling of cardiac electrodynamics^49^. Hence, deep learning has the potential to augment the proposed EEG-based epileptic seizure detection using a feature selection mechanism.

The main contributions in this work are as follows:

(A) A lightweight Deep neural network framework to detect seizures from EEG signals.
(B) Binary version of dragonfly algorithm to select robust and optimal features from the features extracted from the various sub-bands of EEG signals that help to distinguish seizure from non-seizure signals.
(C) Integration of Feature reduction module with DNN to detect the onset of seizure among patients.

The DNN has been proposed to improve the classification accuracy, specificity, and sensitivity, whereas the binary dragonfly algorithm based on swarm intelligence has been used for robust feature selection which helps to improve the detection speed and classifier performance.

## Materials and methods

**Figure 2:**
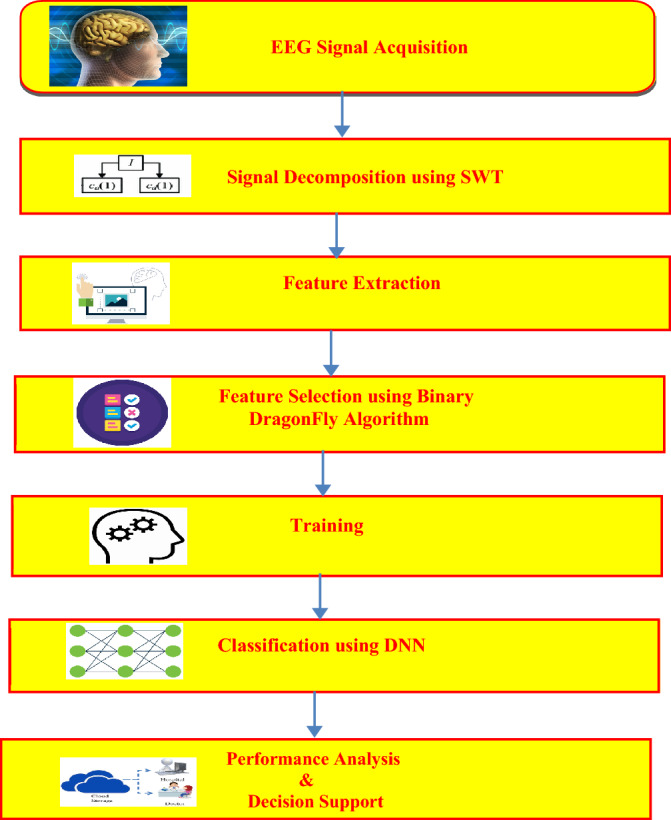
Flow diagram of the proposed Epileptic Seizure Detection system.

The proposed epileptic seizure detection model using the hybrid machine learning-swarm intelligence approach has been shown in Fig. 2. The EEG signals are acquired from the human brain, preprocessed, and applied to Stationary wavelet transform (SWT). The EEG signals have been decomposed into several sub-bands to a level of 4. For each coefficient in each sub-band, the mean absolute value, standard deviation, skewness, kurtosis, RMS power, the ratio of the mean absolute values of adjacent sub-bands, and various Hjorth parameters have been extracted as features. From the extracted features from each sub-band, optimal features have been selected using the binary dragonfly algorithm which is fed as input to the designed DNN model for training. Subsequent to the training phase, the designed model has been used to classify the EEG signals as seizure or normal. Training the DNN using optimal features helps to reduce the overhead incurred by the network and also trains the network quickly. Finally, the performance of the proposed approach has been evaluated in terms of several attributes such as accuracy, sensitivity, specificity, and F1 score. The detailed discussion of the various steps involved in the proposed approach has been discussed in the subsequent sections.

### EEG Signal acquisition and Preprocessing

The BONN-EEG dataset, originating from the University of Bonn, is a versatile resource for EEG signal analysis^26^. The key features include its focus on epilepsy-related research, encompassing both the BONN-EEG Motor Imagery and Epilepsy subsets. These datasets offer multi-subject EEG recordings, with detailed data acquisition specifications like channel count and sampling rate, aiding in experimental design. Annotations indicating events such as seizures in the Epilepsy subset are invaluable for algorithm evaluation. Longitudinal data is also available, allowing researchers to explore changes over time. Various EEG montages cater to diverse research needs, from referential to bipolar, while the dataset's open-access nature fosters collaborative research. Additionally, the dataset often presents challenging characteristics typical of real-world EEG data, such as noise and non-stationarity. Due to its popularity, the BONN-EEG dataset serves as a benchmark for EEG analysis techniques, supporting research in fields like seizure detection, brain-computer interfaces, and neural activity pattern studies.

The open-source Bonn EEG dataset consists of five different sets of data named Sets A, B, C, D, and E. Each set consists of 100 records, whereas each record contains 4097 samples of EEG time series data spanning over 23.6 seconds, captured at a sampling rate of 173.61 Hz under different conditions which are as follows. Set A and B data have been recorded under healthy conditions with eyes open and closed respectively. Set C and D have been recorded from the hippocampal half-sphere area and epileptic area respectively during pre-seizure conditions. Set E has been recorded from epileptic patients under seizure conditions. In this work, for the sake of simplicity and reducing training time we ignored sets B and C for training, and sets A, D, and E have been used for the detection of seizures from the EEG signals. These EEG signals have been preprocessed in two stages: The EEG signals have been passed through a low pass filter for band-limiting them to [0-40Hz] range, checking them for any missing data, and subsequently followed by the normalization of the sample values between the interval [-1,1] for optimum use of the resources and improvement of performance.

### EEG signal decomposition using SWT

**Figure 3:**
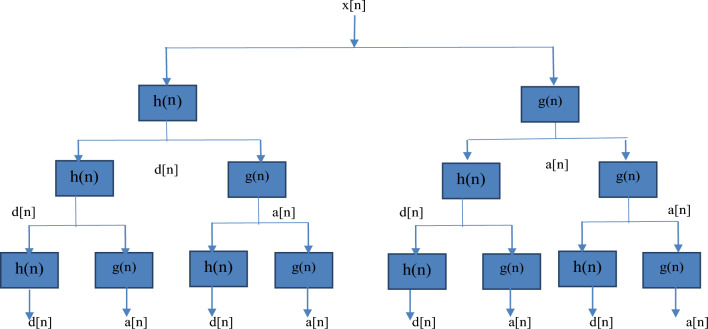
Signal decomposition using SWT.

EEG signals are non-stationary in nature and are effectively characterized in both time domain and frequency domain by using wavelet transform unlike Fourier transform and DCT transforms which can analyze stationary signals only. The wavelet transform considered in this work is of type Discrete Wavelet Transform (DWT) to be more specific Stationary Wavelet Transform. In this stage, the signals have been decomposed into approximation coefficients and detailed coefficients at each level using low-pass and high-pass filters. In general, DWT has been used to preserve high-frequency components, and low-frequency components alone are decomposed at subsequent levels of decomposition. Whereas SWT considers both high and low-frequency components together during the decomposition phase, which provides a lot of insight into the characteristics of EEG signals at different times and different frequencies. A sample decomposition of a signal using SWT is shown in Fig. 3.

**Figure 4:**
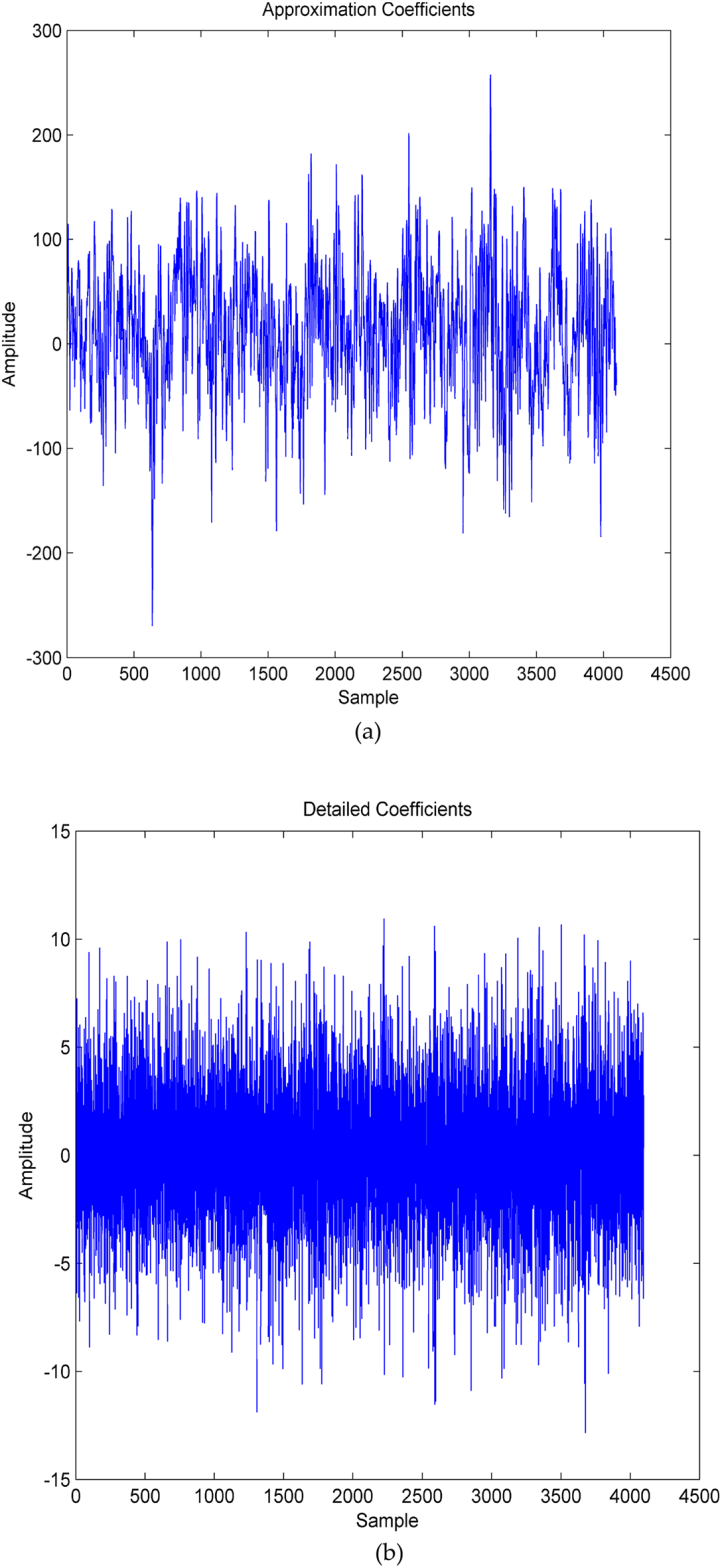
(**a,b**) First level decomposition of normal EEG signal using SWT.

Where h(n) and g(n) refer to high-pass filters and low-pass filters and h(n) and g(n) refers to high-frequency detailed coefficients and low-frequency approximation coefficients respectively. Moreover, SWT has been used in this work to overcome the lack of translation-invariance nature of the DWT by removing the down-samplers and using upsamplers in the DWT. A sample stationary wavelet decomposition of EEG signal using Daubechies-4 wavelet is shown in Fig. 4. In this work, the 'db4' wavelet has been used with the number of levels of decomposition as 4.

The approximation and detailed coefficients of SWT are mathematically represented as given in equation (1)^27^.

1 $$\begin{aligned} & cA_{j,k} (n) = \sum\nolimits_{n} {x(n)\mathop l\nolimits_{j}^{*} } (n - \mathop 2\nolimits^{j} k) \\ & cD_{j,k} (n) = \sum\nolimits_{n} {x(n)\mathop h\nolimits_{j}^{*} } (n - \mathop 2\nolimits^{j} k) \\ \end{aligned}$$

where $$cA_{j,k} (n)$$$$cD_{j,k} (n)$$ and represents the approximation and detailed coefficients respectively. $$l(n)$$ and $$h(n)$$ represents the low pass filter and high pass filter. The variables 'j' and 'k' represent the wavelet scaling and translation factor. The computational complexity and storage requirement of using SWT is $${\rm O}(n\log n)$$.

### Feature extraction

Features are nothing but any distinguishing property, a recognizable measurement, and a functional component extracted from a signal which helps to improve classification accuracy in a classification system. In this work, several statistical features and Hjorth features have been extracted from the decomposed EEG signal for training a classification model. The various features have been used in the proposed model for EEG signal classification are illustrated as follows: Mean absolute value, Standard deviation, skewness, kurtosis, Root mean square (RMS) power, Ratio of the mean absolute values of the coefficients in adjacent sidebands, activity, complexity, and mobility.

1. Mean absolute value is a measure of the average of the absolute sum of the coefficients in each sub-band which is calculated using Eq. (2)^28^.

2 $$\mu =\frac{1}{M}{\sum }_{j=1}^{M}\left|{y}_{j}\right|$$

2. Standard deviation is a measure of the deviation of the coefficients in each sub-band from its mean value and is calculated using Eq. (3)^28^.

3 $$\sigma =\sqrt{\frac{1}{M}{{\sum }_{j=1}^{M}\left({y}_{j}-\mu \right)}^{2}}$$

3. Skewness of the coefficients in each sub-band using Eq. (4)^28^.

4 $$\varphi =\sqrt{\frac{1}{M}{\sum }_{j=1}^{M}\frac{{\left({y}_{j}-\mu \right)}^{3}}{{\sigma }^{3}}}$$

4. Kurtosis of the coefficients in each sub-band. It is a measure of the distribution peaks using the fourth order moment, which is measured using Eq. (5)^28^.

5 $$\varphi =\sqrt{\frac{1}{M}{\sum }_{j=1}^{M}\frac{{\left({y}_{j}-\mu \right)}^{4}}{{\sigma }^{4}}}$$

5. RMS power of the wavelet coefficients in each sub-band using Eq. (6)^28^.

6 $$\lambda =\sqrt{\frac{1}{M}{\sum }_{j=1}^{M}{{y}_{j}}^{2}}$$

6. Ratio of the mean absolute values of adjacent sub-bands using Eq. (7)^28^.

7 $$\chi =\frac{{\sum }_{j=1}^{M}\left|{y}_{j}\right|}{{\sum }_{j=1}^{M}\left|{z}_{j}\right|}$$

7. Activity is a measure of the total power of carried on a signal which is measured by using its variance as shown in Eq. (8)^28^.

8 $$activity=\mathit{var}(y(t))$$

where y(t) represents the signal

8. Mobility is a measure of the first order variations in a signal and it defined as the square root of the ration of variance of first order variation in a signal to the variance of the original signal which is shown in Eq. (9)^28^.

9 $$mobility=\sqrt{\frac{\mathit{var}\left(\frac{dy(t)}{dt}\right)}{\mathit{var}(y(t))}}$$

where dy(t) /dt indicates first-order variation.

9. Signal Complexity is a measure of the level of variations in specific second- order variations along a signal which gives the bandwidth of the signal. It is measured using Eq. (10)^28^.

10 $$Complexity=\frac{mobility(\frac{dy(t)}{dt})}{mobility(y(t))}$$

In this work, we considered data from 300 subjects which are labeled under three categories normal, interictal, and ictal. Each data is decomposed using SWT to four levels and the above features have been calculated. These features are vital for a machine learning algorithm to learn the various characteristics of the EEG signal which in turn classify them into different classes (normal, interictal, ictal).

### Feature selection

Feature selection is meant to improve the accuracy and efficiency of any classifier by selecting the adequate number of features which also helps to reduce the dimension of the problem under consideration. It filters the information that is redundant or unwanted information from the features extracted from the previous phase. In general, three classes of feature selection techniques are common in machine learning which are named wrapper-based, embedded method-based, and filter-based. The feature selection is an NP-hard problem i.e. given 'n' features, the objective is to select an optimal subset of features 'm', where m <  < n which raises $$\left(\begin{array}{c}n\\ m\end{array}\right)$$ combinations. Recently, nature-inspired heuristic algorithms have been more popular among the machine learning research community and are used to solve feature selection problems^34–38^. The performance of various meta-heuristic algorithms in feature selection problems in different domains has been studied and detailed^50^.

In the field of EEG signal classification, an application like epilepsy detection demands robust and efficient feature-reduction techniques to enhance classification accuracy and reduce computational overhead. Hence, the choice of the optimization algorithm plays a pivotal role in determining the effectiveness of feature selection. In the proposed work, Binary dragonfly algorithm (BDFA) has been adopted for selecting the optimal features from the extracted features from EEG signals^38^. The Dragonfly algorithm is a recently evolved metaheuristic swarm intelligence algorithm that has been successfully applied to several continuous optimization problems such as the economic emission dispatch problem, localization problem in networks, various optimization problems in machine learning, etc. BDFA presents a compelling motivation for its use in EEG signal classification and feature reduction. Its compatibility with binary-encoded EEG data, adaptability to dataset characteristics, balance between exploration and exploitation, and competitive performance make it a promising choice for enhancing the accuracy and efficiency of EEG based epileptic seizure detection systems. The BDFA has two phases named exploration and exploitation involved in solving any problem. The BDFA is simple, and it involves a smaller number of parameters and faster convergence to optimal solutions. The apparent randomness in the BDFA behavior is inherent to many nature-inspired optimization algorithms. It allows the algorithm to explore diverse solutions, thereby increasing the likelihood of finding globally optimal or near-optimal solutions in complex problem spaces. Hence, in this work, the optimal feature selection from EEG signal feature space is modeled as a binary optimization problem and solved by using the binary version of the dragonfly algorithm.

The pseudo-code for the BDFA algorithm for feature selection is given in Table 1^37^.

**Table 1:** Pseudocode for Binary DragonFly Algorithm (BDFA).

Initialize the population X_i_ (i = 1, 2, …, n) Initialize ΔX_i_ (i = 1, 2, …, n) Set $\tau_{\min }$ and $\tau_{\max }$ Initialize $\tau = (1 - \frac{t}{T})\tau_{\max } + \frac{t}{T}\tau_{\min }$ While (Termination Criteria) do 1. Evaluate the fitness function of each dragonfly 2. Update Food Source(F) i.e. Best Solution and Enemy(E) i.e. Worst Solution 3. Update the main coefficients (i.,e. w, s, a, c, f, and e) 4. Calculate {$A_{i} = \frac{{\sum\limits_{j = 1}^{N} {V_{j} } }}{N}$, $C_{i} = \frac{{\sum\limits_{j = 1}^{N} {x_{j} } }}{N} - X$, $S_{i} = - \sum\limits_{j = 1}^{N} {X - X_{i} }$, $F_{i} = F_{loc} - X$,$E_{i} = E_{loc} + X$} 5. Update the step vectors using X_i+1_ = X_i_ + ΔX_i+1_ where $\Delta X_{i + 1} = (sS_{i} + aA_{i} + cC_{i} + fF_{i} + eE_{i} ) + wX_{t}$ 6. Calculate T(ΔX) using $$T(\Delta x,\tau ) = \left\{ \begin{gathered} \begin{array}{*{20}c} {1 - \frac{2}{{1 + \mathop e\nolimits^{{\frac{ - 2x}{t}}} }}} & {x \le 0} \\ \end{array} \hfill \\ \begin{array}{*{20}c} {\frac{2}{{1 + \mathop e\nolimits^{{\frac{ - 2x}{t}}} }} - 1} & {x > 0} \\ \end{array} \hfill \\ \end{gathered} \right.$$ 7. Update X_t+1_ using $$x_{i}^{k} (t + 1) = \left\{ \begin{gathered} 1\begin{array}{*{20}c} {} & {rand < T(v_{i}^{k} (t + 1))} & {} \\ \end{array} \hfill \\ 0\begin{array}{*{20}c} {} & {rand \ge T(v_{i}^{k} (t + 1))} & {} \\ \end{array} \hfill \\ \end{gathered} \right.$$ Return the best solution	

A vector of 1's and 0's is used to represent the solution to the feature selection problem, where '0' indicates the corresponding feature is not selected and '1' represents the feature is selected. The fitness function of the feature selection problem is modeled using the classification accuracy and several selected features as given in equation (11).

11 $$Fitness=\alpha {\gamma }_{R}(D)+\beta \frac{\left|C\right|}{\left|N\right|}$$

where α is in the interval [0,1], β = (1 − α), ${\gamma }_{R}(D)$ represents the classification error rate, |C| indicates the number of features selected, and |N| is the total number of features extracted from the EEG signals. In this work, the BDFA parameter settings have been done as follows: $\alpha =0.99$, $\beta =0.01$, population_size = 10, iterations = 100, τ_max_ = 4, τ_min_ = 0.01, s = 0.1, a = 0.1, c = 0.7, f = 1, e = 1, and w = 0.85.

### Training and classification using deep neural networks (DNN)

**Figure 5:**
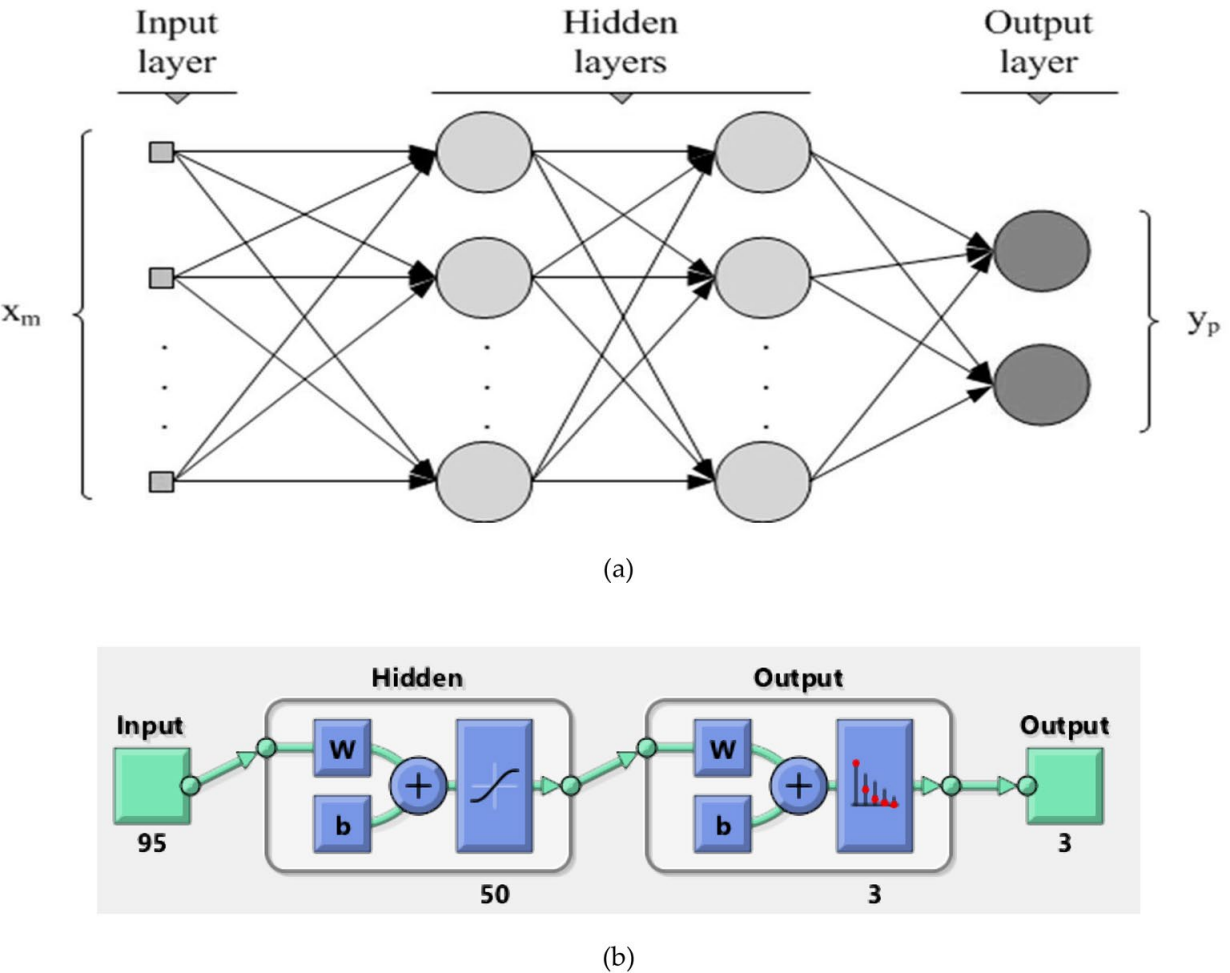
(**a**) Simple Deep Neural Network architecture (with Hidden layers = 2) (**b**) with Hidden layers = 50.

A DNN is an artificial neural network with multiple hidden layers between the input and output layer. The DNN establishes the mathematical relationship between the inputs and outputs which can be either linear or non-linear. DNN with more hidden layers is capable of learning complex functions of the input and it is also characterized by its more abstract representation of data. The DNN classifier builds a multilayer perceptron neural network, which is trained using a set of labeled data which is then validated using a set of unlabeled data to perform classification. A sample DNN model with two hidden layers in between the input layer and output layer is shown in Fig. 5.

The input layer specifies the number of features considered, the output layer specifies the number of classes, and the number of hidden layers determines the architecture of the neural network. In the DNN architecture in Fig. 5, the number of features is 'm' and the number of outputs is 'p'. The network is fully connected and hence each neuron in the previous layer is connected to each neuron in the next layer with a weight 'wij'. The activation function in each neuron maps the weighted sum of inputs to the output of each neuron. The nature of the activation function may vary over different applications and in this work sigmoidal activation function has been used. Learning occurs in the proposed neural network model by changing connection weights between each neuron in an adjacent layer after each chunk of data is processed and based on the amount of error in the anticipated result. Figure 5(a) shows the DNN architecture with 2 hidden layers and Fig. 5(b) shows the DNN architecture with 50 hidden layers.

### Performance analysis and decision support

In this phase, the proposed methodology has been analyzed in terms of several metrics such as classification accuracy, sensitivity, specificity, and F1 score with other related works in seizure detection. The detailed analysis has been discussed in the next section. It is assumed that the proposed system can be trained continuously at regular intervals (hour/day/week/month) which increases the robustness of the system. The trained model shall be deployed in online mode, which increases the detection speed whenever new data is tested in the system and the results will be immediately submitted to medical practitioners through suitable cloud infrastructure for decision support in precision medicine.

## Results and discussion

The proposed hybrid DNN - BDFA methodology for seizure detection has been implemented using Matlab R2020a running in Intel Core-i7 CPU @ 1.90 GHz with 8 GB RAM. The EEG signals from Set A, D, and E have been decomposed using SWT, and the various time domain, frequency domain, and statistical features discussed in "Feature extraction" section were extracted from each sub-band. Though SWT is said to be a redundant transform, building a robust, reliable seizure detection system is essential which provides additional information when compared to DWT towards classification which is indeed truly needed in clinical decision making. From the extracted features, optimal essential features have been selected by applying them to the BDFA, a nature-inspired heuristic algorithm. The selected optimal features have been used to train the DNN model with three hidden layers each with ten neurons constructed using Matlab. The efficacy of the proposed system is tested using 80% of the dataset used for DNN training and validated using the remaining 20% testing dataset. For comparison purposes, we used the various approaches used in^23,29–33^. The performance of the proposed approach is evaluated using the following metrics:

- **Classification Accuracy:** It is defined as the ratio of the number of EEG signals correctly classified to the total number of EEG signals.
  12 $$Accuracy \left(\%\right) =\frac{TP+TN}{TP+FN+FP+TN}\times 100$$
- **Sensitivity:** It is defined as the ratio of True Positives to the total number of actual ictal signals.
  13 $$Sensitivity=\frac{TP}{TP+FN}$$
- **Specificity:** It is defined as the ratio of True Negatives to the total number of actual ictal signals.
  14 $$Specificity=\frac{TN}{TN+FP}$$
  15 $${\text{F1}}\;{\text{Score}} = 2*\left( {{\text{Recall}}*{\text{Precision}}} \right)/\left( {{\text{Recall}} + {\text{Precision}}} \right)$$

**Figure 6:**
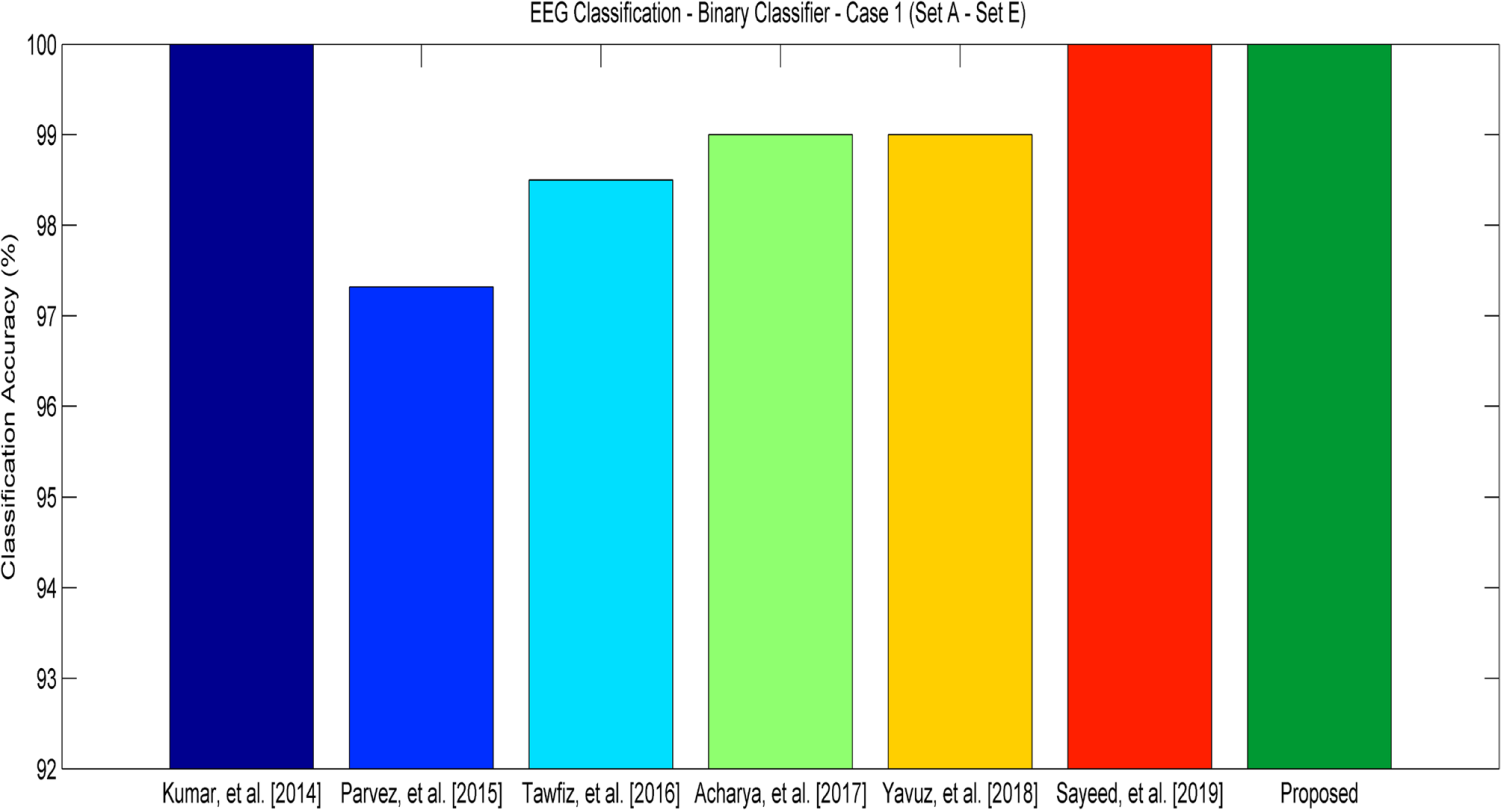
Classification Accuracy of various approaches—Experiment 1 (Set A—Set E).

Three different experiments have been done in this work. The first two experiments have been done as a binary classification problem. The third experiment is done as a multi-label classification problem. In the first experiment, Set A and Set E were used. Set A is considered for non-seizure and Set E is used for Seizure data. In all our experiments 80-20 approach has been used for training, testing and validation. The performance of the proposed approach in terms of accuracy for experiment 1 is shown in Fig. 6. The classification accuracy of the proposed approach is calculated to be 100%, which indicates that all the data are correctly classified as normal and seizure. Even a few of the existing approaches also have the capability to achieve the maximum classification accuracy, they fail to achieve the same in experiment 2 because of the imbalanced dataset.

**Figure 7:**
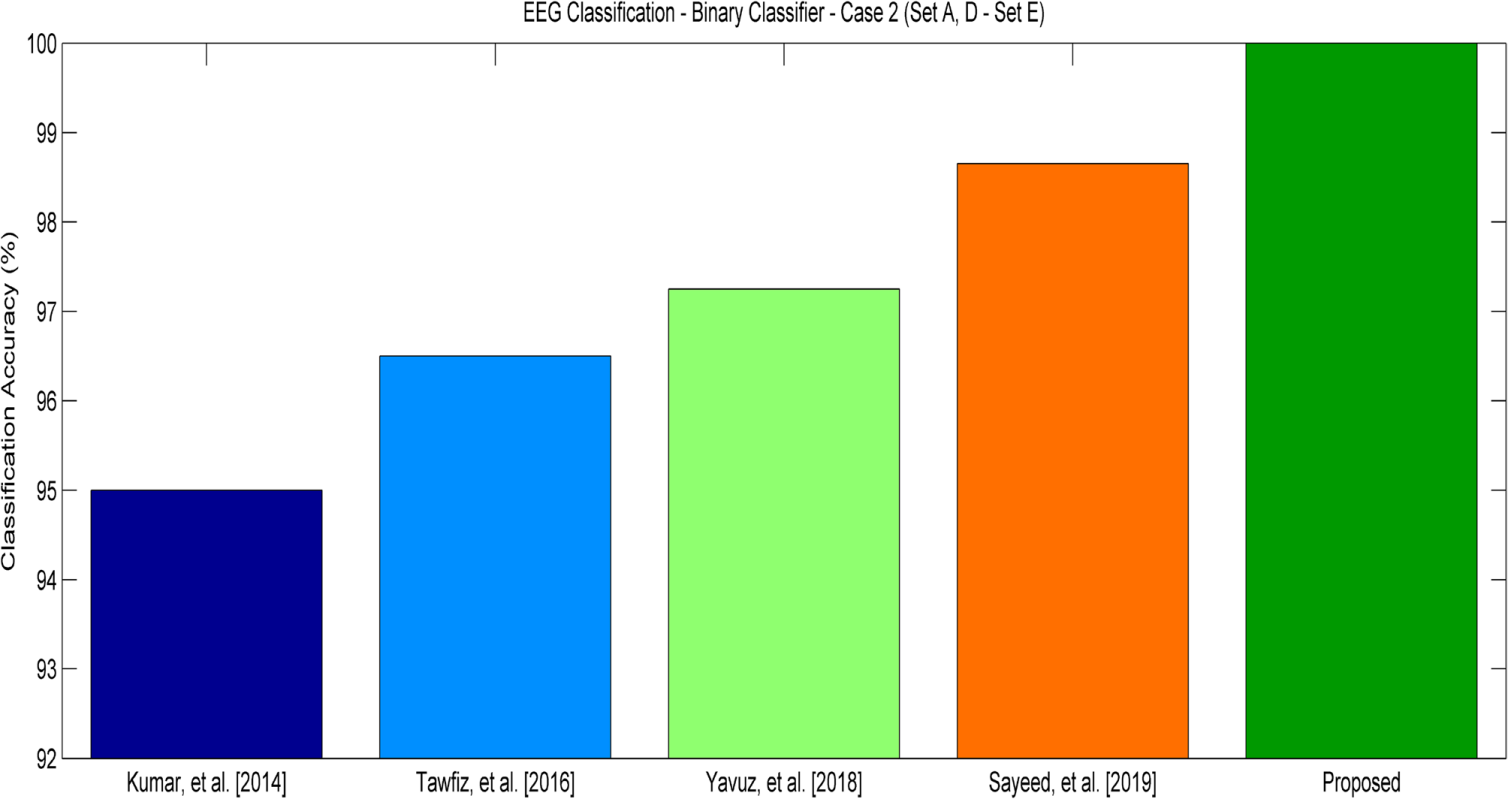
Classification Accuracy of various approaches—Experiment 2 (Set A, D—Set E).

Figure 7 shows the accuracy of experiment 2 using Set A and D data for normal users and Set E for Seizure patients. Though the data is unbalanced with more details for normal users than patients with epilepsy, the proposed SWT equipped with optimal feature selection using BDFA effectively classifies the data accurately than the other existing approaches due to its robust feature selection.

**Figure 8:**
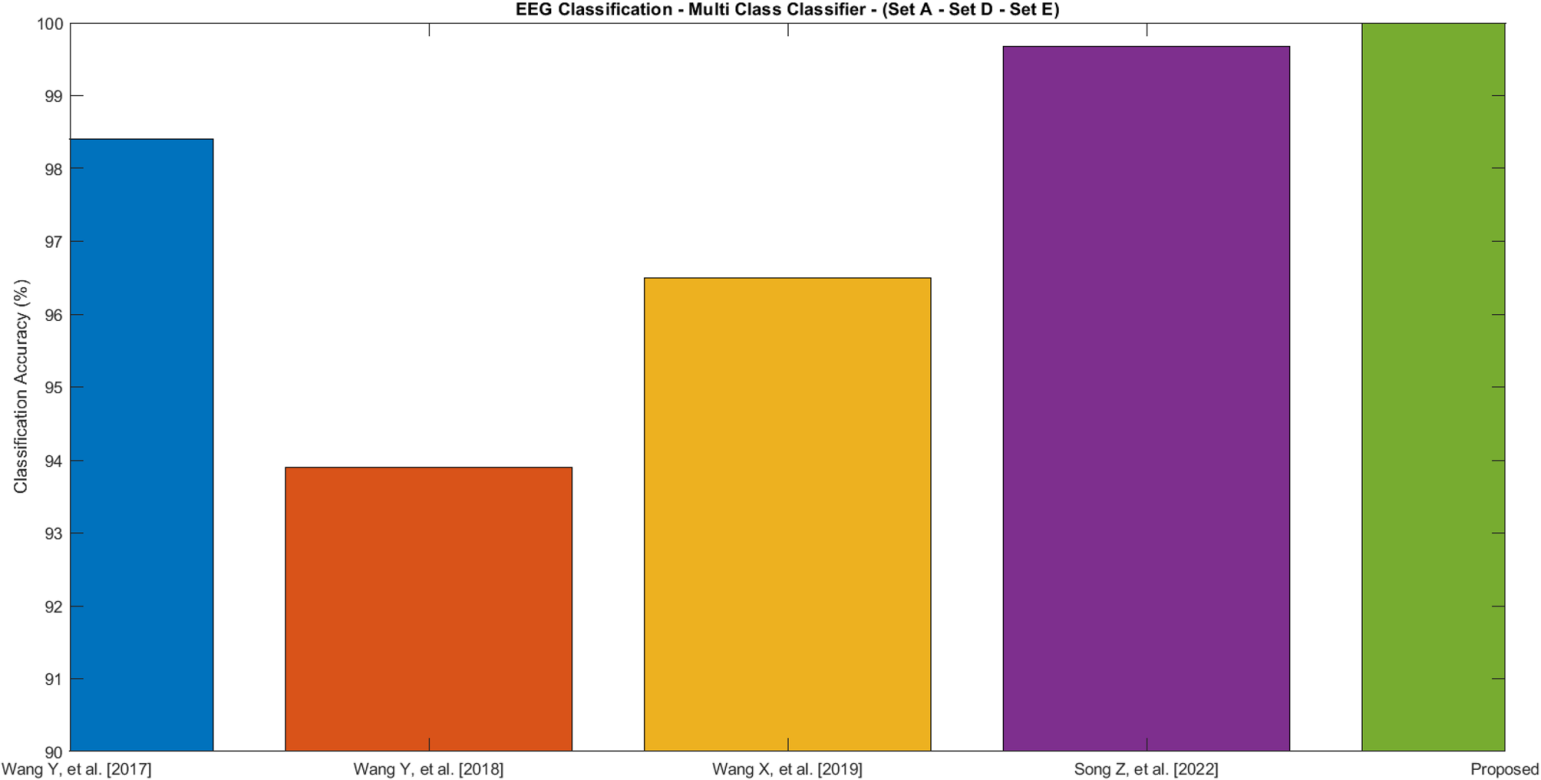
Classification Accuracy of various approaches—Experiment 3 (Set A—Set D—Set E).

Figure 8 shows the performance of the multi-label classifier problem using three datasets named Set A, D, and E. The three corresponding labels are normal, interictal, and ictal. Most of the existing approaches are able to achieve accuracy around 94-99%, the proposed model is able to achieve 100% accuracy by labeling all the instances correctly. The sensitivity, specificity and F1-score are also evaluated to be 100% in the proposed approach. This will help the medical practitioners to correctly identify the conditions of the patients with utmost confidence and subsequently followed by suitable medications if needed. By using the infrastructures associated with IoT, the proposed model helps the medical practitioners assess the conditions of patients remotely, which can save a significant amount of time.

Table 2 provides a subset of various features mentioned in "Feature extraction" section that have been extracted from the three classes of EEG signals considered in this work. The average, minimum, maximum, and standard deviation values of the various features in each set is calculated and tabulated in Table 2. It is evident that, from most of the features that have been extracted it is easy to classify the signal as seizure or normal or interictal. However, whether all the features that has been Extracted is really needed for accurate classification is really a big question. Using all the features for classification is always good, but it consumes more time during training and testing. Hence, the BDFA has been used to select the minimal number of optimum features that are well enough to provide better classification performance.

**Table 2:** A subset of features Extracted from the EEG dataset.

Features/category		Mean absolute value (MAV)	Standard deviation	Skewness	Kurtosis	RMS Power	MAV ratio between adjacent sidebands	Activity	Mobility	Complexity
Normal Signal	MAX	106.7859	78.94465	0.218029	4.326632	111.7333	0.790126	6232.258	0.545033	2.604474
	MIN	27.65216	31.65529	−0.35486	2.746312	34.91518	0.707107	1002.057	0.247076	1.455799
	AVG	54.16027	57.37346	−0.02148	3.230966	66.64868	0.727363	3427.589	0.360995	1.972086
	STD	16.22131	11.71526	0.114972	0.27586	16.89996	0.016739	1301.82	0.06438	0.235525
Interictal Signal	MAX	384.4638	622.3303	2.875449	14.44804	622.3172	0.718972	387,295	0.297324	4.668411
	MIN	22.75521	27.17553	−1.95023	2.620492	29.19894	0.702985	738.5096	0.123272	1.712706
	AVG	76.35908	92.71864	0.076662	4.309593	99.99496	0.709926	15,214.5	0.206204	2.823581
	STD	55.39758	81.75938	0.758032	2.571865	80.30411	0.002848	44,354.38	0.043508	0.558374
Ictal Signal (Seizure)	MAX	711.3554	866.1592	2.255783	9.70101	866.0678	0.758486	750,231.8	0.557975	3.525137
	MIN	114.7202	134.8554	−1.57528	1.930258	149.6405	0.696656	18,185.99	0.173916	1.191911
	AVG	345.724	433.0973	−0.05962	3.401424	435.4889	0.716124	230,797.8	0.327848	1.788587
	STD	164.5184	208.9524	0.752165	1.179565	207.4267	0.010699	201,320.6	0.075077	0.40559

Regarding feature selection, it is the question of selecting the minimal number of features from a set of 143 features with 300 instances. For each decomposed signal using SWT at each level, all the tabulated features have been calculated which leads to a total of 143 features i.e. (16×9-1). The BDFA algorithm has been applied to the extracted feature set which selects the minimal number of features selected using KNN classifier is with 10-fold cross-validation is used to evaluate the performance of the selected feature subset. The performance of the feature selection using BDFA is measured in terms of the selected feature ratio and Fischer ratio. Selected feature ratio is defined as the ratio of the number of features selected to the total number of extracted features. In this work, the selected feature ratio is calculated to be 19/143= 0.13 which helps to ensure high classification speed and accuracy.

Fisher ratio is a measure of how far the data points in different classes are separated and how close the data points in similar classes are evaluated over the selected subset of features^38^. The average Fisher score is evaluated to be 0.08 using equation 16^38^.

16 $${F}_{Tot}=\frac{1}{S}{\sum }_{i=1}^{S}{F}_{i}$$

where S refers to the number of features in the subset and Fi refers to Fischer index for each feature.

Table 3 summarizes the performance of the proposed algorithm in terms of various performance metrics. The results indicate that the proposed approach achieves significant improvement in performance with reduced complexity by minimizing the features during the classification phase. The proposed approach uses just 13% of available features to achieve 100% accuracy, precision, specificity, sensitivity and F1 score when compared to other existing studies.

**Table 3:** Performance Comparison with existing studies – Summary.

Metric	Performance (%)			
	Yavuz et al. (2018)	Sayeed et al. (2019)	Song et.al(2022)	Proposed
Accuracy	97.25	98.65	**99.67**	**100**
Precision	97.20	99.10	**99.67**	**100**
Sensitivity	97	97.30	99.67	100
Specificity	96.70	98.3	99.67	100
F1 score	97.10	98.20	99.67	100
Selected feature ratio	100	100	**100**	**13**

The strength of the proposed approach is its simplicity and being light-weight. The DNN learns from the robust features selected using the BDFA algorithm to classify a EEG signals as seizure or normal which may help to quick emergency response on demand. Though the BDFA is simple and robust and offers several advantages for EEG signal feature selection, it's essential to consider the potential limitations such as computational overhead for large datasets, careful parameter tuning, risks of overfitting, and convergence speed. Careful study of the BDFA and datasets helps to improve the performance irrespective of various limitations mentioned above in real-time classification and feature selection problems. Also, the proposed method will be tested against other benchmarks datasets on EEG signals to detect epileptic seizures in future and to develop edge IoT device to identify seizure and to initiate medication immediately on demand.

## Conclusion

In this paper, an improved epileptic seizure detection system using deep neural network and binary dragonfly algorithm has been proposed. Stationary wavelet transform is used to decompose EEG signals into different sub-bands and various features are extracted from them. The binary dragonfly algorithm is used to select the robust and optimal features that are sufficient enough to detect the condition of seizure has been selected. The deep neural network model was then trained using the robust features selected using the nature-inspired heuristic algorithm and used the knowledge base to classify the ictal and interictal EEG signals from normal EEG signals. The heuristic algorithm selects a robust feature subset of extracted features which improves the classification accuracy and speeds up the training and detection process. The experimental results illustrate that the proposed approach achieves 100% classification accuracy, sensitivity, specificity, F1 score, and average Fischer score with just a 13% selected feature ratio when compared to existing approaches. This proposed system helps medical practitioners to diagnose and heal epileptic patients at a higher rate by integrating it with hospitals using the Internet of Medical Things which can provide precision medicine. In future, we planned to construct an edge device that detects the seizure onset condition and alert the medical practitioners and family members to initiate the medications and also to test the efficacy of the proposed approach on several EEG datasets.

## Data Availability

The datasets used during the current study are available from the corresponding author on reasonable request.
